# Correction to article ‘The mechanics behind DNA sequence-dependent properties of the nucleosome’

**DOI:** 10.1093/nar/gkab464

**Published:** 2021-05-25

**Authors:** Eugene Y D Chua, Dileep Vasudevan, Gabriela E Davey, Bin Wu, Curt A Davey

**Affiliations:** School of Biological Sciences, Nanyang Technological University, 60 Nanyang Drive, 637551, Singapore and NTU Institute of Structural Biology, Nanyang Technological University, 59 Nanyang Drive, 636921, Singapore; School of Biological Sciences, Nanyang Technological University, 60 Nanyang Drive, 637551, Singapore and NTU Institute of Structural Biology, Nanyang Technological University, 59 Nanyang Drive, 636921, Singapore; School of Biological Sciences, Nanyang Technological University, 60 Nanyang Drive, 637551, Singapore and NTU Institute of Structural Biology, Nanyang Technological University, 59 Nanyang Drive, 636921, Singapore; School of Biological Sciences, Nanyang Technological University, 60 Nanyang Drive, 637551, Singapore and NTU Institute of Structural Biology, Nanyang Technological University, 59 Nanyang Drive, 636921, Singapore; School of Biological Sciences, Nanyang Technological University, 60 Nanyang Drive, 637551, Singapore and NTU Institute of Structural Biology, Nanyang Technological University, 59 Nanyang Drive, 636921, Singapore

The Authors wish to alert Readers to an error in their article (1). The error is shown in bold below, and can be found in the Results section, under ‘Minor groove-inward regions dominate sequence-dependent attributes’, on page 6344.

‘…Nonetheless, the optimal major groove-inward sequences consist mostly or purely of G|C nucleotides and the ideal motifs apparently correspond to alternating dinucleotide types, wherein the flexibility inherent **in CG and GC steps** is seemingly stabilizing…’

Should read

‘…Nonetheless, the optimal major groove-inward sequences consist mostly or purely of G|C nucleotides and the ideal motifs apparently correspond to alternating dinucleotide types, wherein the flexibility inherent **in CG steps** is seemingly stabilizing…’

This error does not affect the results or conclusions of the article.
